# Impact of organised cervical screening on cervical cancer incidence and mortality in migrant women in Australia

**DOI:** 10.1186/1471-2407-12-491

**Published:** 2012-10-23

**Authors:** Nayyereh Aminisani, Bruce K Armstrong, Sam Egger, Karen Canfell

**Affiliations:** 1 School of Public Health, University of Sydney, Sydney, Australia; 2 Cancer Council New South Wales, Sydney, Australia; 3 Faculty of Health and Nutrition, Tabriz University of Medical Sciences, Tabriz, Iran

## Abstract

**Background:**

Organised cervical screening, introduced in 1991, appears to have reduced rates of cervical cancer incidence and mortality in women in Australia. This study aimed to assess whether cervical cancer rates in migrant women in the state of New South Wales (NSW) showed a similar pattern of change to that in Australian-born women after 1991.

**Methods:**

Data from the NSW Central Cancer Registry were obtained for females 15+ years diagnosed with invasive cervical cancer from 1973 to 2008 (N=11,485). We used joinpoint regression to assess annual percent changes (APC) in cervical cancer incidence and mortality before and after the introduction of organised cervical screening in 1991.

**Results:**

APC in incidence fell more rapidly after than before 1991 (p<0.001) amongst women from seven groups defined by country of birth (including Australia). There was only weak evidence that the magnitude of this incidence change varied by country-of-birth (p=0.088). The change in APC in mortality after 1991, however, was heterogeneous by country of birth (p=0.004). For Australian and UK or Ireland-born women the mortality APC fell more rapidly after 1991 than before (p=0.002 and p=0.001 respectively), as it did for New Zealand, Middle East, North Africa and Asian-born (p≥0.05), but in other European-born and women from the ’Rest of the World’ it appeared to rise (p=0.40 and p=0.013 respectively).

**Conclusions:**

Like Australian-born women, most, but not all, groups of migrant women experienced an increased rate of fall in incidence of cervical cancer following introduction of organised cervical screening in 1991. An apparent rise in mortality in women in a ‘Rest of the World’ category might be explained by a recent rise in migration from countries with high cervical cancer incidence and mortality rates.

## Background

Incidence of and mortality from cervical cancer have generally fallen following the establishment of organised cervical screening programs in developed countries [1]. Since the introduction of organised screening in Australia in 1991, cervical cancer incidence and mortality rates among women 20 years of age and older have fallen substantially [2,3], by about 50% to date [4].

Cervical cancer remains one of the most common cancers in women in developing countries, and has its highest incidence in women in Sub-Saharan Africa, Central America, South-Central Asia, and Melanesia [5]. Studies in various populations with well organised screening programs have also documented disparities in cervical cancer incidence and mortality between women born overseas and native-born women [6-12]. In New South Wales (NSW), the most populous Australian state, cervical cancer incidence among some migrant groups, notably women born in Vietnam or Fiji, is higher than in Australian-born women [13-15]. It has been suggested that variation in cervical screening uptake or in treatment may explain these disparities. The impact of organised screening programs on the incidence and mortality of cervical cancer, however, has not been assessed in different migrant groups in any country. Thus it is not known whether women of different origins have shared equally in the benefits of organised cervical screening.

The aim of this study was to assess whether migrant women in Australia shared with Australian-born women the downturn in cervical cancer incidence and mortality that was observed following the 1991 introduction of organised cervical screening.

## Methods

Data for this study were obtained from the NSW Central Cancer Registry (CCR), which was established in 1972 [16]. The CCR is a population-based registry supported by a statutory obligation for all public and private hospitals, radiotherapy facilities, nursing homes, outpatient departments and day-procedure centres to notify malignant neoplasms [16].

Women of all ages who were diagnosed with, or died from, invasive cervical cancer in the period 1973 to 2008 and who had complete information on age and country of birth were included in these analyses (10,820 incident cases and 4,037 deaths). We obtained data on country of birth, date of diagnosis, age at diagnosis, and date of death. To calculate incidence rates, the mid-year estimated resident female population for NSW by 5 year age group and by country of birth (COB) over the period of study was obtained from the Australian Bureau of Statistics (ABS) and from the Health Outcomes Information Statistical Toolkit (HOIST) which is a 'data warehouse' operated by the Centre for Epidemiology and Research of the NSW Department of Health. Annual population estimates by sex, age and COB were not available before 1981, so we used estimates from the Census populations of 1972, 1976 and 1981 for Census years and two years either side of these years (as needed) for annual values [17]. To correctly estimate the female population at risk of cervical cancer, the proportion of women who had undergone hysterectomy prior to each year of study should be considered and removed from the population for that year. However, we did not perform this correction in the main analyses because hysterectomy frequencies by calendar year, age and region of birth were only collected by the NSW Admitted Patient Data Collection from 1991 onwards. In order to assess whether this had an impact on our findings we performed sensitivity analysis in which hysterectomy frequencies were modelled by fitting a generalised linear model, assuming a Poisson distribution and a log link function, to the NSW Admitted Patient Data from 1991 to 2008. In the sensitivity analysis we identified no substantial differences between the results derived from the original analyses and those derived from the hysterectomy-corrected analyses (see Appendix).

The NSW Population and Health Services Research Ethics Committee approved this project. De-identified data were used for the analysis.

### Statistical analysis

Age was grouped into 5-year age groups for age-adjustment in Poisson regression and in three categories (20–49 years, 50–69 years, and 70+ years) for the analysis of age-specific trends. Country of birth was grouped into 7 regions [18]: Australia, New Zealand (NZ), the United Kingdom and Ireland, rest of Europe, the Middle East and North Africa, Asia, and the ‘Rest of the World’. These groups were based on numbers in different country of birth categories from the 2006 Census [19] and formed to create a manageable number of regions of birth with reasonably large numbers and reasonable cultural homogeneity.

We used generalised linear models, assuming a Poisson distribution and a log link function, to compare the annual percent change (APC) in cervical cancer incidence and mortality rates from 1973 to 1991 and from 1991 to 2008. Comparisons were performed for all NSW cervical cancer cases and deaths and in subgroups defined by region of birth and three broad age groups: 20–49 years, 50–69 years, and 70+ years. The dependent variable in each model was either the number of newly diagnosed cervical cancer cases or the number of cervical cancer deaths for each combination of categories of the independent variables; with the corresponding mid-year populations included as an offset. The independent variables included categories of age (<20 years, 20–24, 25–29…, 80–84, 85+ years), region of birth (Australia, New Zealand (NZ), the United Kingdom and Ireland, rest of Europe, the Middle East and North Africa, Asia, and the ‘Rest of the World’), calendar year of diagnosis (1973–2008 as a continuous variable) and calendar year of diagnosis or death for the period after the introduction of organised screening (1992–2008 or zero for years prior to 1992, also a continuous variable). The last of these independent variables and its corresponding regression function parameter allows a potential change in the APC occurring after 1991 to be modelled through a join-point [20]. Terms for the interaction between regions of birth, broad age group and calendar year of diagnosis or death variables were included where appropriate to allow APCs to be estimated separately for each subgroup. To account for possible over-dispersion, Poisson standard errors were inflated by a scale parameter equal to the Pearson chi-squared statistic divided by the residual degrees of freedom [21].

For graphical presentation the observed age-adjusted cervical cancer incidence and mortality rates were plotted against the fitted age-adjusted rates derived from the regression models. Observed age-adjusted rates were calculated in the usual manner using observed age-specific rates and the 1991 Australian Standard Population. Fitted age-adjusted rates were calculated using the expected age-specific rates predicted by the estimated regression functions. To assess whether our results were robust to the choice of statistical method, we also performed the same analyses using join-point weighted linear regression models in which the log of the age-adjusted rate was regressed against the calendar year of diagnosis or death. Observations were weighted by the inverse of the variance of the log of the age-adjusted rate and errors were assumed to be auto-correlated with lag one (the use of this method to analyse trends in age-adjusted rates is described in detail elsewhere [22]). Because the results derived from the weighted linear regression were almost identical to those derived from the Poisson regression, we report only the results from the Poisson regression. We consider the Poisson method to have a slight technical advantage for analysing the current data because of its ability to handle calendar years with zero cases or deaths whereas the weighted linear regression method requires the introduction of a small correction factor [23]. All data were analysed using STATA version 11 (STATA Corp, Texas USA).

## Results

### Descriptive characteristics of the study population

A total of 11,485 women were diagnosed with cervical cancer between 1973 and 2008 in NSW. Of these, information on country of birth and age was available for 7,635 Australian-born women and 3,185 overseas-born (migrant) women (Table 1). The mean ages of the Australian-born and migrant women included in the analyses were broadly similar at 52.2 (SD=16.8) years and 53.9 (SD=15.4) years respectively.

**Table 1:** Distributions by age and country of birth of women diagnosed with and dying from cervical cancer in NSW in 1973-2008

	***Incidence***		**Mortality**	
	**N**	**%**	**N**	**%**
**Age**				
<20	16	0.15	1	0.02
20-29	656	6.1	64	1.6
30-39	2118	19.6	322	8.0
40-49	2247	20.8	582	14.4
50-59	1920	17.7	742	18.4
60-69	1914	17.7	922	22.8
70+	1949	18.0	1404	34.8
**Country of birth**				
Australia	7635	70.6	2953	73.1
NZ	203	1.9	52	1.3
UK& Ireland	946	8.7	384	9.5
Rest of Europe	1035	9.6	401	9.9
Middle East & North Africa	144	1.3	40	1.0
Asia	599	5.5	132	3.3
Rest of the World	258	2.4	75	1.9

There were 4,049 women who died of cervical cancer between 1973 and 2008 in NSW; of these, information on country of birth and age was available for 2,953 Australian-born women and 1,084 overseas-born women (Table 1). The mean ages of Australian-born and overseas-born women dying from cervical cancer were broadly similar at 61.6 (SD=15.9) years and 62.5 (SD=15.4) years, respectively.

**Figure 1:**
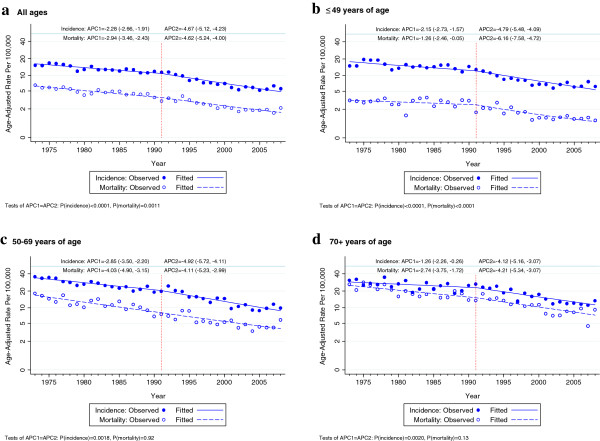
Trends in age-adjusted cervical cancer incidence and mortality by broad age categories in NSW women, 1973–2008(APC1: annual percent change from 1973-1991, APC2: annual percent change from 1991-2008).

There was a highly significant difference in the annual percent changes (APC) in cervical cancer incidence and mortality when rates before 1991 were compared with those after the introduction of organised screening in 1991 in NSW women of all ages and countries of birth (Figure 1, Table 2). For incidence, the APC was −2.28 up to, and −4.67 after, 1991 (p<0.001) and for mortality it was −2.94 up to, and −4.64 after 1991 (p=0.001).

**Table 2:** Annual percent change (APC) in age-adjusted cervical cancer incidence and mortality by region of birth and broad age categories before and after organised screening in NSW

**Age group**	**Region of birth**	***APC (95%CI) incidence trends***			***APC (95%CI) mortality trends***		
		**Up to organised screening**	**After organised screening**	***P value***	**Up to organised screening**	**After organised screening**	***P value***
**All ages**							
	**All regions**	−2.28(−2.66,-1.91)	−4.67(−5.12, -4.23)	<0.0001	−2.94(−3.46,-2.43)	−4.62(−5.24,-4.00)	0.0011
	**Australia**	−2.25(−2.68,-1.81)	−4.81(−5.34,-4.27)	<0.0001	−2.80(−3.39,-2.20)	−4.64(−5.37,-3.90)	0.0024
	**New Zealand**	−1.60(−4.58,1.46)	−5.06(−7.95,-2.08)	0.20	−0.09(−5.26,5.37)	−4.42(−9.15,0.56)	0.35
	**United Kingdom/Ireland**	−1.86(−3.09,-0.61)	−4.14(−5.68,-2.57)	0.073	−2.28(−3.87,-0.66)	−7.91(−10.16,-5.60)	0.0014
	**Rest of Europe**	−3.40(−4.57,-2.21)	−3.47(−4.93,-1.99)	0.95	−4.68(−6.30,-3.03)	−3.30(−5.22,-1.33)	0.40
	**Middle East/North Africa**	−2.36(−5.72,1.12)	−8.49(−11.99,-4.85)	0.050	0.34(−6.52,7.69)	−2.99(−8.06,2.37)	0.55
	**Asia**	−1.45(−3.69,0.84)	−6.39(−7.91,-4.85)	0.0033	−0.85(−5.04,3.52)	−6.52(−9.26,-3.71)	0.069
	**Rest of the World**	−2.45(−5.48, 0.67)	−0.39(−2.82, 2.11)	0.41	−7.51(−11.70,-3.13)	2.11(−1.92, 6.31)	0.013
**≤49 years of age**							
	**All regions**	−2.15(−2.73,-1.57)	−4.79(−5.48,-4.09)	<0.0001	−1.26(−2.46,-0.05)	−6.16(−7.58,-4.72)	<0.0001
	**Australia**	−2.11 (−2.75, -1.47)	−5.00 (−5.78, -4.21)	<0.0001	−0.80 (−2.17, 0.60)	−6.16 (−7.80, -4.50)	<0.0001
	**New Zealand**	−3.42 (−7.33, 0.65)	−3.77 (−7.56, 0.17)	0.92	−1.92 (−11.54, 8.74)	−4.76 (−13.37, 4.70)	0.74
	**United Kingdom/Ireland**	0.34 (−1.84,2.57)	−3.48(−5.99, -0.90)	0.074	3.90 (−1.23, 9.29)	−12.60 (−19.03, -5.66)	0.0020
	**Rest of Europe**	−1.52 (−3.38, 0.36)	−4.09 (−6.81, -1.30)	0.22	−4.60 (−8.02, -1.04)	−2.23 (−7.55, 3.40)	0.56
	**Middle East/North Africa**	−5.76 (−10.26, -1.03)	−9.96 (−16.17, -3.30)	0.40	15.21 (−3.09, 36.97)	−27.35 (−43.13, -7.19)	0.0092
	**Asia**	−3.67 (−6.74, -0.49)	−4.45 (−6.69, 2.16)	0.74	−4.15 (−11.54, 3.86)	−4.79 (−10.27, 1.04)	0.91
	**Rest of the World**	−1.26 (−5.57,3.25)	−0.05 (−3.40, 3.41)	0.73	−10.06 (−17.43, -2.04)	1.80 (−6.50, 10.83)	0.12
**50-69 years of age**							
	**All regions**	−2.85(−3.50,-2.20)	−4.92(−5.72,-4.11)	0.0018	−4.03(−4.40,-3.15)	−4.11(−5.23,-2.99)	0.92
	**Australia**	−2.88 (−3.62, -2.14)	−5.04 (−6.00, -4.06)	0.0056	−3.91 (−4.92, -2.90)	−4.11 (−5.45, -2.76)	0.85
	**New Zealand**	2.41 (−3.80, 9.03)	−8.61(−14.27,-2.58)	0.046	1.86 (−8.60, 13.52)	−1.05 (−9.34, 7.99)	0.75
	**United Kingdom/Ireland**	−3.89 (−5.86, -1.88)	−4.75 (−7.44,-2.00)	0.69	−4.34 (−7.17, -1.43)	−7.83 (−12.13, -3.32)	0.29
	**Rest of Europe**	−5.61 (−7.34, -3.85)	−3.57 (−5.75, -1.33)	0.26	−5.90 (−8.49, -3.24)	−4.11 (−7.44, -0.66)	0.51
	**Middle East/North Africa**	2.84 (−3.57, 9.68)	−9.13 (−13.99,-4.01)	0.019	−5.63 (−16.11, 6.16)	4.39 (−4.54, 14.16)	0.29
	**Asia**	0.26 (−3.30, 3.97)	−8.88 (−11.31, -6.38)	0.0007	1.80 (−5.27, 9.40)	−9.59 (−14.18, -4.75)	0.031
	**Rest of the World**	−4.69 (−9.43, 0.30)	−1.14 (−5.28, 3.18)	0.40	−3.45 (−10.84, 4.54)	0.22 (−5.73, 6.53)	0.56
**70+ years of age**	**All regions**	−1.26(−2.26,-0.26)	−4.12(−5.16,-3.07)	0.0020	−2.74(−3.75,-1.72)	−4.21(−5.34,-3.07)	0.13
	**Australia**	−1.27 (−2.37, -0.14)	−4.11 (−5.30, -2.91)	0.0065	−2.75 (−3.92, -1.57)	−4.19 (−5.51, -2.84)	0.21
	**New Zealand**	−1.88 (−9.04, 5.85)	−4.37 (−12.37, 4.35)	0.73	0.47 (−9.22, 11.19)	−12.94 (−25.31, 1.47)	0.20
	**United Kingdom/Ireland**	−1.67 (−4.09, 0.81)	−4.19 (−7.17,-1.11)	0.31	−2.42 (−4.99, 0.21)	−6.55 (−10.04, -2.94)	0.14
	**Rest of Europe**	−3.78 (−7.29, -0.14)	−2.26 (−5.46, 1.06)	0.62	−2.70 (−6.50, 1.25)	−3.65 (−7.00, -0.17)	0.77
	**Middle East/North Africa**	−2.44 (−11.98, 8.13)	−8.79 (−17.36, 0.66)	0.46	−2.49 (−15.91,13.08)	−1.58 (−11.69, 9.70)	0.94
	**Asia**	9.27 (−0.66, 20.19)	−9.39 (−13.44, -5.16)	0.0026	0.36 (−10.20, 12.17)	−4.50 (−10.30, 1.67)	0.52
	**Rest of the World**	0.59 (−9.91, 12.32)	−1.66 (−8.99, 6.26)	0.79	−10.83 (−20.67,0.24)	4.86 (−5.73, 16.62)	0.12

**Figure 2:**
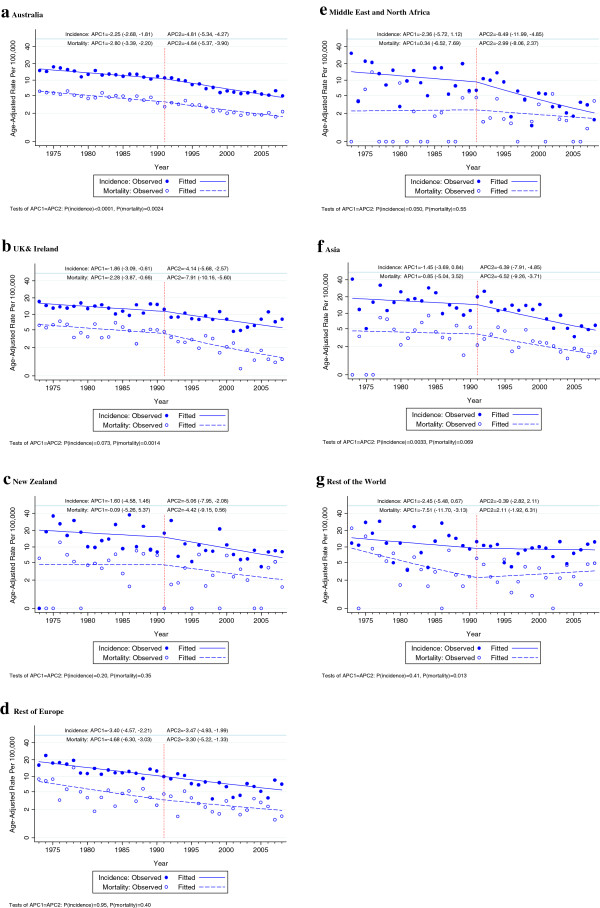
Trends in age-adjusted cervical cancer incidence and mortality in all ages by region of birth in NSW women, 1973–2008APC1: annual percent change from1973-1991, APC2: annual percent change from 1991-2008.

These patterns in the APC in cervical cancer incidence before and after 1991 were also seen in women with different countries of birth (Figures 2a-g and Table 2), except in women born in Europe (other than in UK or Ireland), who showed a steady downtrend in incidence across the two periods, and in women born in the ‘Rest of the World’, who showed almost no fall in incidence after 1991, although with a wide confidence interval. Patterns in the APC in cervical cancer mortality were generally similar to those in incidence except that for women born in the Rest of the World mortality appeared to increase after 1991. The incidence and mortality P-values for heterogeneity among regions of birth in the change in slope after 1991 were 0.088 and 0.004, respectively.

The overall trends in incidence up to and after 1991 were also evident in the APCs in incidence for the broad age categories 20–49 years, 50–69 years and 70+ years (p≤0.002 in each case; Figures 1b-d and Table 2) and in the APC in cervical cancer mortality for women aged <49 years (p<0.0001; Figure 1d). In older women, there was little difference in the APC in mortality up to and after 1991. The incidence and mortality P-values for heterogeneity among age groups in the change in slope after 1991 were 0.75 and 0.005 respectively. Examination of trends by age and region of birth showed that a greater fall in the APC in cervical cancer rates after 1991 than before was evident in most age and country of birth categories (Table 2). At 40–49 years of age, it was seen for incidence in six of seven regions of birth and for mortality in five of seven. At 50–69 years of age it was seen for incidence in five of seven and for mortality in four of seven and at 70+ years of age for incidence in 6 of 7 and in mortality for five of seven.

## Discussion

As has been reported previously [2,3], organised cervical screening from 1991 was apparently effective in increasing the rate of fall in cervical cancer incidence and mortality in NSW women. We observed this pattern in both incidence and mortality in women born in Australia, New Zealand, the United Kingdom and Ireland, Asia and the Middle East and North Africa. For women born in Europe (other than the United Kingdom and Ireland) and the ‘Rest of the World’, however, there was no evidence of an increase in the rate of fall in incidence after 1991 and for women born in the Rest of the World mortality from cervical cancer may have increased after 1991.

While we did not attempt to relate the observed downtrends up to 1991 to change in cervical screening during this period, opportunistic screening and better treatment probably increased following the introduction of universal health insurance in 1975 with reimbursement of the cost of cervical cytology and access free-of-charge to treatment of cervical cancer precursors and cervical cancer itself. Changes in sexual behaviour might also have influenced these and the later trends. There is evidence from studies both in and outside Australia that mortality from cervical cancer in young women was increasing just before or early in the study period [24-27] due, perhaps, to the changes in sexual mores that occurred during and after the Second World War. These changes might have kept cervical cancer incidence and mortality rates higher in the first part of the period than they would otherwise have been. It is possible, also, that increasing condom use after the beginning of the epidemic of HIV infection during the 1980s [28] could have contributed to the downtrends in the 1990s. In addition, it is possible that the observed trends may have been influenced by differential exposure over time and between various groups of migrant women to the established co-factors of human papillomavirus (HPV) infection (parity, age at first full term pregnancy, use of tobacco and oral contraceptives) The very small fall in incidence and the increase in mortality from cervical cancer in women from the Rest of the World after 1991 may reflect recent changes in patterns of migration to Australia. Many women classified as from the ‘Rest of the World’ have come from African and Latin America countries where cervical cancer is very common [5]. The numbers of migrants from these areas have increased in the last two decades [29,30]. Thus the apparently less favourable trend in incidence and the unfavourable trend in mortality in this migrant group may reflect the high cervical cancer incidence and mortality of their home region, which would be expected to persist for some time after coming to Australia.

No previous study has examined trends in cervical cancer incidence and mortality in migrant women in Australia. While a number of international studies in diverse populations have documented disparities in cervical cancer incidence and mortality between migrant women and native-born women or women of different ethnic backgrounds [6-12], only a few have examined time trends in cervical cancer incidence or mortality by ethnic background or country of birth.

A New Zealand study [31] examined trends in cancer incidence from 1981–1986 to 2002–2004 in four groups defined by ethnicity or country of birth: Maori, Pacific Islander, Asian and European or Other. The investigators found that, compared to European or Other women, Maori, Pacific Islander, and Asian women had higher incidence rates of cervical cancer in 2002–2004. The age standardised incidence rate ratio (SRR) comparing Maori and Pacific Islander women with European/Other women fell with time, which suggests a greater rate of fall in Maori and Pacific Islander than European/Other women. However, the SRR for Asian women increased with time. While New Zealand introduced organised cervical screening in 1991, this report did not compare trends before and after this date.

McDougall et al. [6] examined trends in incidence of cervical cancer by ethnicity in the US between 1992 and 2003 based on information from the 13 cancer registries. They found similar falls in incidence of cervical cancer overall and in squamous cell carcinoma in four different ethnic groups: Non-Hispanic whites, Hispanic whites, African-American, and Asian or Pacific Islander, over the period of study. However falls were more pronounced among Asian or Pacific Islanders. A second US study [8], examined cervical cancer incidence trends in four categories: Hispanic/all races, Non-Hispanic/white, non-Hispanic/black, non-Hispanic/other using a dataset from 22 state cancer registries. Incidence of cervical cancer was significantly less in all four race/ethnic groups in 2000–2004 than 1995–1999 (rate ratio 0.83, 95% CI 0.82-0.84), with standardized rate ratios ranging from 0.75 (95% CI 0.70-0.79) for Non-Hispanic/other to 0.84 (95% CI 0.82-0.85) for non-Hispanic/white. A third US study examined the incidence and mortality of cervical cancer among Asian and non-Hispanic white women in California in 1990 to 2004. Cervical cancer incidence and mortality fell in each group during this period. The APCs in the rates were −8.7% for Vietnamese, -5.1% among Koreans, -4.6% among Filipinos, -5.4% for Chinese and −2.3% among non-Hispanic Whites [10]. None of these studies related the trends in incidence or mortality with trends in cervical screening, which is largely opportunistic in the USA.

When examined in broad age categories, there was less evidence in older than younger NSW women that mortality fell faster after 1991 than it did up to 1991. This age group difference could be due to less screening [32-34] or less effective screening in older women [35] after 1991, although the first should also affect incidence trends. We have no reason to think that older women treated for cervical pre-neoplasia or cancer would have received poorer treatment after 1991 than before. However, it is important to note that the optimal interval for screening is longer in women over 50 years [1] when compared to younger women, and it has previously been proposed that apparently greater effects of opportunistic screening in older women could be partly due to the greater efficacy of irregular screening in older than younger women [3].

Our findings are based on 36 years of data from a high quality cancer registry covering a large population. The period for which cancer registry data was available included 18 years before the year of introduction of organised cervical screening program (1991) and 17 years after; thus trends before and after introduction of an organised approach could be modelled with considerable precision in a range of groups of migrant women.

We analysed trends in cervical cancer rates in relation to country of birth, but we did not have individual-level data on the age of migration, and therefore could not explicitly account for prior screening experience in the country of origin. However, cytological screening in women aged 20–24 years has been shown to have little or no impact on rates of invasive cervical cancer up to age 30 years [36], and therefore any effects of pre-migration screening for women who migrated as children or young adults are expected to be very limited. Even for women who migrated at ages older than 25 years, the results of a major audit and case–control study in the UK (a study which underpins IARC’s 2005 recommendations for the cervical screening interval) [1], found that the relative risk of invasive cancer in screened women dropped to the same level as that of unscreened women after 3.5 years in women younger than 50 years, and after 5–6 years in women over 50 years of age [37]. In effect, the cytological screening effect ‘wears off’ relatively rapidly which is why frequent repeated screening is required with cytology. Therefore, it is unlikely that screening history before migration would have a major impact on the trends observed in this study.

We were unable to adjust for hysterectomy over the whole analysis period because hysterectomy frequencies by calendar year, age and region of birth were only collected by the NSW Admitted Patient Data Collection from 1991 onwards. However, we conducted sensitivity analyses using the NSW Admitted Patient Data to correct the populations at risk for hysterectomies and found very similar results to those in the main analysis. In addition, the age adjusted hysterectomy incidence rates in women 20-85+ years of age, estimated from the NSW Admitted Patient Data Collection [38], were stable from 1991 to 1997 and then fell steadily in all country of birth groups from 5.2/1,000 (Australian born) to 3.0/1,000 (Asian born) in1997 to a half to two-thirds of the 1997 values (3.1/1,000 in Australian born to 1.7/1,000 in Asian born) in 2008 (data not shown). The effect of these trends would be to reduce the observed rate of fall in cervical cancer rates in all country of birth groups in the later part of the period following introduction of organised screening. This effect, though, would be greater in older than younger women and thus might comprise part of the explanation of the less evident increase in the downtrends in cervical cancer incidence and mortality in older women after 1991 (Figures 1b-d).

We were, in addition, not able address possible confounding of trends by country of birth by, for example, trends by socioeconomic status and area of residence. Information on socioeconomic status based on the Australian Bureau of Statistics Index of Relative Socioeconomic Disadvantage (IRSD) was not available from the CCR for years before 1980 and classification of area of residence based on Accessibility and Remoteness Index for Areas (ARIA) was only available from 2000. Neither of these, however, would be expected to have had much of a confounding effect. There were only small differences in the distributions of IRSD between Australian-born and migrant women studied from 1980 and the vast bulk of both Australian born and migrant women studied from 2000 resided in major city or inner regional areas (89.8% and 98.0% respectively).

Finally, it should be noted that rates of invasive cervical cancer incidence and mortality in Australia nationally appear to have stabilised since about 2002 [39]; reductions prior to that time have been predominately driven by declines in invasive squamous cervical cancer, whereas the incidence of adenocarcinoma (glandular cancers) appears not to have been substantially impacted by cytological-based screening. This stabilisation effect may have resulted in our calculated average annual percent changes from 1991 to 2008 being slightly lower than if rates had not stabilised over the last 5 years of the period included in our assessment. However, in this study we have taken a broad approach to assessing the overall changes in rates after the period of interest, and focused mainly on the differentials between various groups of migrant women. We have identified some groups of migrant women from certain European and other countries that may be able to benefit, to a greater degree, from the organised cervical screening program in Australia. In the context of a current review of cervical screening recommendations and technology in Australia [40], further reductions in invasive cervical cancer incidence and mortality rates could also potentially be achieved in the population overall by switching to a primary screening test which is potentially more effective in detecting adenocarcinoma, such as primary HPV DNA-based screening. In the longer term, the National HPV Vaccination Program, introduced in Australia in 1997, also has the potential to further reduce rates of cervical cancer in all Australian women [41].

## Conclusions

As has been the case for Australian-born women, most categories of migrant women experienced an increased rate of fall in incidence of cervical cancer following introduction of organised cervical screening in 1991. Most, but not all, also experienced an increased rate of fall in cervical cancer mortality. An apparent rise in mortality in women in a ‘Rest of the World’ category may be explained by a recent rise in migration from countries with high cervical cancer incidence and mortality rates.

## Appendix. Sensitivity analysis for the effect of hysterectomy rates

To correctly estimate the female population at risk of cervical cancer, women who had undergone hysterectomy should be excluded from the population at risk for that year. However, we did not perform this correction in the main analyses because hysterectomy frequencies by calendar year, age and region of birth were only collected by the NSW Admitted Patient Data Collection from 1991 onwards. However, we conducted sensitivity analyses using the NSW Admitted Patient Data to correct the populations at risk for the effect of hysterectomy. Hysterectomy frequencies were modelled using methods specified in detail elsewhere [42]. In brief, this involved fitting a generalised linear model assuming a Poisson distribution and a log link function to the NSW Admitted Patient Data from 1991 to 2008. The dependent variable in the model was the number of new hysterectomies for each combination of categories of the independent variables with the corresponding mid-year populations included as an offset. The independent variables included categories of age (20–24, 25–29…, 80–84, 85+ years; it was assumed that hysterectomies did not occur at <20 years of age), region of birth (Australia, New Zealand (NZ), the United Kingdom and Ireland, rest of Europe, the Middle East and North Africa, Asia, and the rest of the world), calendar year of diagnosis (1991–2008 as a continuous variable) and 18 birth cohorts (1888-1978+ as continuous variable with linear and quadratic terms). Age, year of diagnosis and birth cohort effects were modelled specific to each region of birth through the inclusion of appropriate interaction terms. The estimated regression equation was then used to predict hysterectomy rates within and beyond the range of observed values of the independent variables so as to provide predicted hysterectomy rates for the diagnosis years 1973–2008. For each region of birth, year of diagnosis and birth cohort, the predicted rates were converted to cumulative probabilities of having an intact uterus and cervix at age *x* using the cumulative risk formula *P*_*x*_ = exp(*Σ*_*i* = 1_^*x*^ − 5*r*_*i*_) where *P*_*x*_ is the cumulative probability at age *x* and r_i_ is the predicted hysterectomy rate at age *i*. This formula accounts for the sequential removal of women who have previously had hysterectomies from the population at risk of the procedure [42,43]. Hysterectomy-corrected populations at risk were then calculated by multiplying the cumulative probabilities of having an intact uterus and cervix by the corresponding all-women populations. The main analyses of cervical cancer trends were then re-executed after replacing the all-women populations with the hysterectomy-corrected populations.

Additional file 1: Figure S1 indicates that the modelled age-standardised hysterectomy rates (using 1991 Australian Standard Population) were good predictors of the empirical age-standardised rates for the years of diagnosis for which data were available (1991–2008). Additional file 1: Figures S2(a-c) and S3(a-g) show that no substantial differences were observable between the results derived from the original analyses and those derived from the hysterectomy-corrected analyses.

## Competing interests

The authors declare that they have no competing interests.

## Authors’ contributions

Authors’ contribution: All authors were involved in design of the protocol and preparation of the Human Research Ethics Committee application. NA and SE were responsible for data analysis and SE performed the sensitivity analysis. NA and SE prepared drafts of the manuscript. BA and KC supervised and supported data analysis. All authors contributed to all drafts of the manuscript, including the final one. All authors read and approved the final manuscript.

## Pre-publication history

The pre-publication history for this paper can be accessed here:

http://www.biomedcentral.com/1471-2407/12/491/prepub

## Supplementary Material

Additional file 1: Figure S1 Trends in age standardised hysterectomy rates by region of birth in NSW women, 1973–2008. Modelled and empirical rates using hysterectomy-corrected populations as denominators. **Figure S2- **Trends in age-adjusted cervical cancer incidence and mortality by broad age categories in NSW women, 1973–2008. Fig S2a All ages. Fig S2b ≤49 years of age. Fig S2c 50–69 years of age. Fig S2d 70+ years of age. **Figure S3- **Trends in agecorrected cervical cancer incidence and mortality in all ages by region of birth in NSW women, 1973–2008. Fig S3a Australia. Fig S3b UK& Ireland. Fig S3c New Zealand. Fig S3d Rest of Europe. Fig S3e Middle East and North Africa. Fig S3f Asia. Fig S3g Rest of the world. Click here for file
