# Supplementary material for: Impact of organised cervical screening on cervical cancer incidence and mortality in migrant women in Australia
Source: BMC Cancer. 2012 Oct 23;12:491. doi: 10.1186/1471-2407-12-491 (PMC3573959; doi:10.1186/1471-2407-12-491)
Supplement: Additional file 1: Figure S1 — Trends in age standardised hysterectomy rates by region of birth in NSW women, 1973–2008. Modelled and empirical rates using hysterectomy-corrected populations as denominators. Figure S2- Trends in age-adjusted cervical cancer incidence and mortality by broad age categories in NSW women, 1973–2008. Fig S2a All ages. Fig S2b ≤49 years of age. Fig S2c 50–69 years of age. Fig S2d 70+ years of age. Figure S3- Trends in agecorrected cervical cancer incidence and mortality in all ages by region of birth in NSW women, 1973–2008. Fig S3a Australia. Fig S3b UK& Ireland. Fig S3c New Zealand. Fig S3d Rest of Europe. Fig S3e Middle East and North Africa. Fig S3f Asia. Fig S3g Rest of the world. [file 1471-2407-12-491-S1.doc]

## Figure S1: Trends in age standardised hysterectomy rates by region of birth in NSW women, 1973-2008. Modelled and empirical rates using hysterectomy-corrected populations as denominators.

## Figure S2- Trends in age-adjusted cervical cancer incidence and mortality by broad age categories in NSW women, 1973-2008

(APC1: annual percent change from 1973-1991, APC2: annual percent change from 1991-2008)

Fig S2a All ages

^1^Original results shown in Figure 1a in the manuscript repeated here for convenience. ^2^Corrected results derived using the same models to generate Figure 1a in the manuscript but using hysterectomy-corrected populations at risk.

Fig S2b ≤49 years of age

^1^Original results shown in Figure 1b in the manuscript repeated here for convenience. ^2^Corrected results derived using the same models to generate Figure 1b in the manuscript but using hysterectomy-corrected populations at risk.

Fig S2a 50-69 years of age

^1^Original results shown in Figure 1c in the manuscript repeated here for convenience. ^2^Corrected results derived using the same models to generate Figure 1c in the manuscript but using hysterectomy-corrected populations at risk.

Fig S2b 70+ years of age

^1^Original results shown in Figure 1d in the manuscript repeated here for convenience. ^2^Corrected results derived using the same models to generate Figure 1d in the manuscript but using hysterectomy-corrected populations at risk.

## Figure S3- Trends in age-corrected cervical cancer incidence and mortality in all ages by region of birth in NSW women, 1973-2008

(APC1: annual percent change from 1973-1991, APC2: annual percent change from 1991-2008)

Fig S3a Australia

^1^Original results shown in Figure 2a in the manuscript repeated here for convenience. ^2^Corrected results derived using the same models to generate Figure 2a in the manuscript but using hysterectomy-corrected populations at risk.

Fig S3b UK& Ireland

^1^Original results shown in Figure 1a in the manuscript repeated here for convenience. ^2^Corrected results derived using the same models to generate Figure 1b in the manuscript but using hysterectomy-corrected populations at risk.

Fig S3c New Zealand

^1^Original results shown in Figure 1a in the manuscript repeated here for convenience. ^2^Corrected results derived using the same models to generate Figure 1b in the manuscript but using hysterectomy-corrected populations at risk.

Fig S3d Rest of Europe

^1^Original results shown in Figure 1a in the manuscript repeated here for convenience. ^2^Corrected results derived using the same models to generate Figure 1b in the manuscript but using hysterectomy-corrected populations at risk.

Fig S3e Middle East and North Africa

^1^Original results shown in Figure 1a in the manuscript repeated here for convenience. ^2^Corrected results derived using the same models to generate Figure 1b in the manuscript but using hysterectomy-corrected populations at risk.

Fig S3f Asia

^1^Original results shown in Figure 1a in the manuscript repeated here for convenience. ^2^Corrected results derived using the same models to generate Figure 1b in the manuscript but using hysterectomy-corrected populations at risk.

Fig S3g Rest of the world

^1^Original results shown in Figure 1a in the manuscript repeated here for convenience. ^2^Corrected results derived using the same models to generate Figure 1b in the manuscript but using hysterectomy-corrected populations at risk.
